# Genetic and morphological differentiation in *Populus nigra* L.: isolation by colonization or isolation by adaptation?

**DOI:** 10.1111/mec.13192

**Published:** 2015-05-14

**Authors:** Jennifer DeWoody, Harriet Trewin, Gail Taylor

**Affiliations:** Centre for Life Sciences, Unviersity of SouthamptonBuilding 85, Highfield Campus, Southampton, SO17 1BJ, UK

**Keywords:** biomass, common garden experiment, European black poplar, leaf size, microsatellites

## Abstract

Identifying processes underlying the genetic and morphological differences among populations is a central question of evolutionary biology. Forest trees typically contain high levels of neutral genetic variation, and genetic differences are often correlated with geographic distance between populations [isolation by distance (IBD)] or are due to historic vicariance events [isolation by colonization (IBC)]. In contrast, morphological differences are largely due to local adaptation. Here, we examined genetic (microsatellite) and morphological (from a common garden experiment) variation in *Populus nigra* L., European black poplar, collected from 13 sites across western Europe and grown in a common garden in Belgium. Significant genetic differentiation was observed, with populations from France displaying greater admixture than the distinct Spanish and central European gene pools, consistent with previously described glacial refugia (IBC). Many quantitative traits displayed a bimodal distribution, approximately corresponding to small-leaf and large-leaf ecotypes. Examination of nine climatic variables revealed the sampling locations to have diverse climates, and although the correlation between morphological and climatic differences was significant, the pattern was not consistent with strict local adaptation. Partial Mantel tests based on multivariate summary statistics identified significant residual correlation in comparisons of small-leaf to large-leaf ecotypes, and within the small-leaf samples, but not within large-leaf ecotypes, indicating that variation within the small-leaf morphotype in particular may be adaptive. Some small-leaf populations experience climates very similar to those in large-leaf sites. We conclude that adaptive differentiation and persistent IBC acted in combination to produce the genetic and morphological patterns observed in *P. nigra*.

## Introduction

Identifying the evolutionary processes controlling genetic structure and morphological diversity is a central aim of evolutionary biology. The genetic structures of species have been examined using putatively neutral genetic markers for decades (Brown 1978; Loveless & Hamrick 1984; Hamrick & Godt 1996). Genetic differentiation at neutral markers is typically driven by gene flow between populations, genetic drift and mutation of novel alleles (Wright 1931). Natural selection is not expected to act on neutral markers; thus, genetic structure should reflect demographic histories, that is the level of inbreeding and migration within and among populations. In plant species, the mating system and dispersal mechanism have significant effects on the levels of genetic differentiation observed among populations (Loveless & Hamrick 1984; Duminil *et al*. 2007). Historic vicariance events may also affect the distribution of genetic variation, especially in longer-lived species (Nason *et al*. 2002; Petit *et al*. 2003; Savolainen & Pyhajarvi 2007).

Plant morphology, in contrast, is widely considered adaptive (Turesson 1922; Westoby & Wright 2006). Phenotypic differentiation among populations reflects a balance between natural selection in the local environment, migration of alleles via gene flow (Antonovics 1968) and, at a lower frequency, the acquisition of novel traits through mutation. Natural selection within a population must be strong enough to overcome gene flow from morphologically divergent populations in order to maintain phenotypic differentiation (Kremer *et al*. 2012). The ever-increasing literature comparing differentiation in morphological traits and that at neutral marker loci indicate that phenotypic differentiation is typically greater than neutral genetic differentiation, implying natural selection overcomes ongoing gene flow to maintain morphological differences (Merila & Crnokrak 2001; McKay & Latta 2002; Cavers *et al*. 2004; Steane *et al*. 2006; Hall *et al*. 2007; Leinonen *et al*. 2013).

The consequences of these adaptive and neutral processes are not mutually exclusive, and natural populations are expected to experience them in combination (Cavers *et al*. 2004; Orsini *et al*. 2013). Divergent phenotypic selection may drive genetic differentiation at neutral loci (Nosil *et al*. 2009). Significant structure is expected at neutral loci if selection is sufficient to reduce the fitness of maladapted migrants (LeCorre & Kremer 2003; Nosil *et al*. 2009). Over generations, multilocus genotypes at neutral loci may become differentiated as a result of selective pressures. While correlations between allele frequencies and environment have been reported in several plant species (e.g. Kelly *et al*. 2003; Mitton & Duran 2004; Sork *et al*. 2010), isolation by adaptation (IBA), the correlation between allele frequencies and morphology while controlling for geographic separation, is rarely considered in studies across larger geographic areas. Recent literature reviews identified up to 33 studies addressing IBA in genetic differentiation, with only two focusing on tree species (Nosil *et al*. 2009; Orsini *et al*. 2013).

The well-established and widely tested process of isolation by distance (IBD) results in genetic differentiation increasing as a function of the geographic distance between populations (Slatkin 1993; Rousset 1997). That is, populations that are more distant geographically will have lower rates of gene flow, and will differentiate even in the absence of divergent selection. This contemporary IBD has recently been called isolation-by-dispersal limitation (Orsini *et al*. 2013) Geographic structure may result in morphological divergence due to genetic drift alone (Dennison & Baker 1991; Eckert *et al*. 1996).

For many species, an extreme geographic isolation took place at the most recent glacial maximum (ending 15 000 years before present), which was sufficient to reduce gene flow among refugia across the Northern Hemisphere. This historic vicariance resulting from subsequent postglacial migration, called isolation by colonization (IBC), is distinct from contemporary IBD and may produce different patterns of genetic differentiation (Spurgin *et al*. 2014). If contemporary levels of gene flow and the time since colonization are sufficient, the genetic signatures of IBC may be eroded, leaving no relationship between geographic distance and genetic differentiation. Serial colonization, however, is expected to produce a persistent correlation between genetic and geographic distance at both neutral and adaptive loci (Orsini *et al*. 2013), even in the absence of local adaptation. Recolonization patterns following glacial melting have been well described in forest trees using chloroplast markers (Kremer *et al*. 2002; Petit *et al*. 2002; Palme *et al*. 2003; Cottrell *et al*. 2005). While historic genetic signatures are well studied, morphological differentiation among recolonized populations is rarely considered a consequence of historic phenotypes of the ancestral gene pools. Rather, even in long-lived forest trees, phenotype is expected to be a consequence of local adaptation (i.e. correlate to climatic differences and not geographic distance) (Gailing *et al*. 2012), and not reflect the historic differences (Kremer *et al*. 2002).

*Populus nigra* L., the European black poplar, is a riparian tree widely distributed across Europe and into Asia and northern Africa. Likely restricted to three glacial refugia in western Europe during the most recent glacial maximum (Cottrell *et al*. 2005), chloroplast haplotype patterns identified two major and one minor routes taken by *P. nigra* in recolonizing central Europe. Populations in France were most likely colonized from the Iberian refuge, while populations in Germany, the Lowlands, and eastward into central Europe probably arose from refugia in the Italian peninsula and Balkan region. Today, *P. nigra* is recognized as both ecologically and economically important in Europe. As a keystone species, *P. nigra* colonizes newly disturbed riparian sites and provides critical habitat in these human-impacted environments. Due to the modification of major rivers across Europe and its natural patchy preferred habitat, *P. nigra* is restricted to isolated populations across the landscape. As a fast-growing tree, *P. nigra* is the focus of breeding and management programmes for wood and biofuel production aimed at identifying genetic variants involved in growth (Fabbrini *et al*. 2012).

Across its range, *P. nigra* displays remarkable phenotypic variation. Trees from central Europe display high growth rates, large central stems and large leaf sizes, and consequently high biomass production. Black poplar trees from regions having hot and dry Mediterranean summers produce smaller leaves and a branching growth habit, likely partial adaptations to seasonal drought (Viger 2011). A better understanding of the evolution of morphological variation and genetic structure in *P. nigra* will assist breeding and conservation efforts.

This study tested for correspondence between genetic structure, morphological variation and climatic differences in *P. nigra* genotypes sampled from 13 populations from western Europe and grown in a common garden study. We tested for IBD and IBA by comparing neutral genetic markers, phenotypic traits and climate differences between sampling sites. Genetic differentiation was examined using a panel of nine microsatellite markers, and morphological variation was assessed for 12 traits in the common garden study. We then used multivariate analyses and full and partial Mantel tests to examine the relative roles of geographic and adaptive isolation in determining the genetic structure of a keystone forest tree.

## Materials and methods

### Study system and collections

**Fig. 1:**
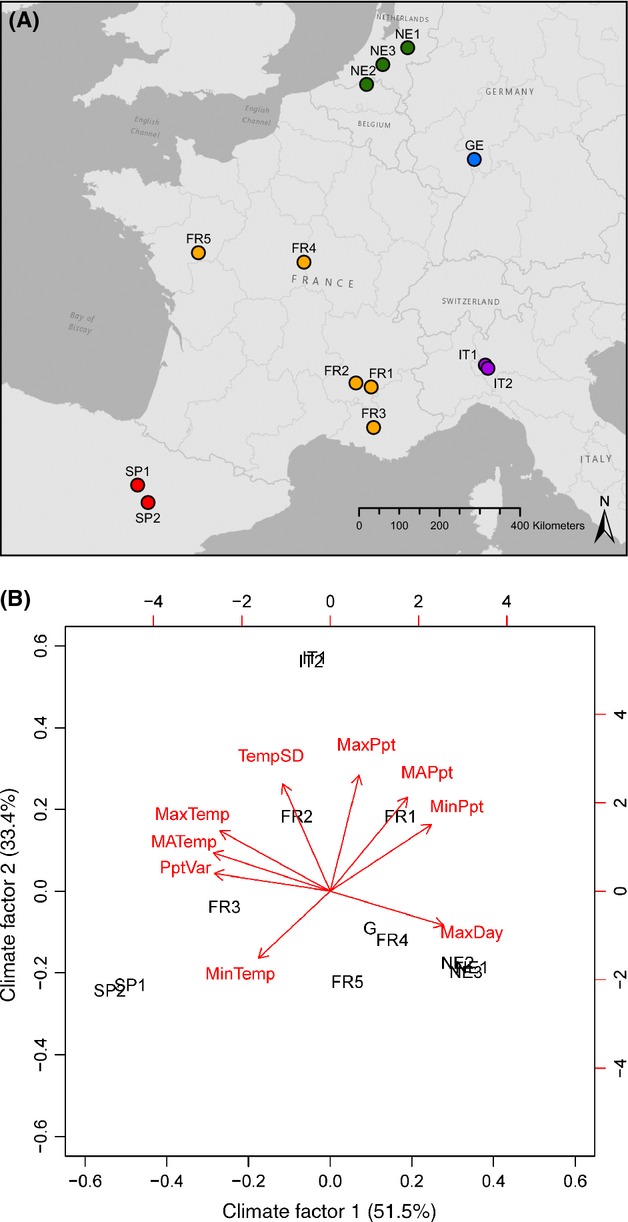
(A) Locations of *Populus nigra* populations sampled in Spain (red), France (orange), the Netherlands (green), Germany (blue) and Italy (purple) for the common garden study. Abbreviations follow Table 1. (B) Principal components analysis of nine climatic variables (following Table 2) reveal heterogeneous environments at the 13 sampling locations. Red arrows and text indicate the loadings of each climate variable, while black text indicates the relative climate of each sampling site.

*Populus nigra* L. is restricted to riparian habitat, resulting in a patchy occurrence across the broadest landscape scale. Unlike the canonical climax forest tree species, *P. nigra* is fast-growing, early successional, and dioecious, with male and female flowers occurring on separate plants. Samples were collected from 13 natural populations of *P. nigra* from France, Germany, Italy, the Netherlands and Spain (Rohde *et al*. 2011; Table 1, Fig.1). To describe the environmental differences experienced by each population, a variety of meteorological data was gathered for each collection site: four measures of temperature: mean annual temperature (MATemp, °C), temperature seasonality (standard deviation ×100, SDTemp), maximum temperature of the warmest month (MaxTemp, °C) and minimum temperature of the coldest month (MinTemp, °C); four measures of precipitation: mean total annual precipitation (MAPpt, mm), precipitation seasonality (a coefficient of variation; VarPpt), precipitation of the wettest month (MaxPpt, mm) and precipitation of the driest month (MinPpt, mm); and the maximum day length on the summer solstice (MaxDay, h). Temperature and precipitation data were taken from http://www.worldclim.org (Hijmans *et al*. 2005), a collection of data from the years 1950 to 2000. Maximum day length was from the U.S. Naval Observatory Astronomical Applications Department (http://aa.usno.navy.mil/data/docs/RS_OneDay.php) and served as a proxy for latitude.

**Table 1:** *Populus nigra* populations sampled for phenotypic analysis in a common garden study

Abbrev.	Country	Population	Latitude	Longitude	*N*_M_	*N*_G_
FR1	France	Drôme 1	44.6833	5.4000	63	31
FR2	France	Drôme 6	44.7500	4.9167	63	45
FR3	France	Durance	43.7847	5.5569	12	9
FR4	France	Loire East	47.3295	2.9200	26	6
FR5	France	Loire West	47.2571	−0.5870	21	13
GE	Germany	Kuhkopf	49.8167	8.5000	56	44
IT1	Italy	La Zelata	45.2667	8.9833	63	41
IT2	Italy	Siro Negri wood	45.2000	9.0667	44	32
NE1	The Netherlands	Ijssel, Rhine	52.2250	5.9600	31	23
NE2	The Netherlands	Individual clones	51.3583	4.5500	7	5
NE3	The Netherlands	Waal, Maas	51.8194	5.0900	12	9
SP1	Spain	Ebro 1	41.9333	−1.3833	54	20
SP2	Spain	Ebro 2	41.5833	−1.000	60	30

Latitude and longitude provided in dd.dddd.

*N*_M_ = number of genets analysed for morphological traits, 512 in total.

*N*_G_ = number analysed for genetic markers, 308 in total.

To identify the most informative but least correlated variables from the climate data set, a principal component analysis (PCA) was conducted as a means of data reduction as implemented in the r programing language (version 3.0.2) using the package stats. The first two climate components were compared to morphological (discriminant factors) and genetic (principal coordinates) differences among populations. Correlation between climatic differences and geographic distances between populations was examined using Mantel tests as implemented in genalex v. 6 (Peakall & Smouse 2006).

### Microsatellite analyses: resolving neutral genetic structure

A subset of genotypes from each population (*N* = 308 in total) were analysed for neutral nuclear markers (Table 1). Leaves from each genotype in the common garden experiment were sampled, flash frozen in liquid nitrogen and stored at −80 °C until processed. DNA extraction and microsatellite analyses followed standard procedures as detailed in the Methods S1 (Supporting information). A panel of nine biparentally inherited, codominant microsatellite markers was assayed for each sample: PMGC_14, PMGC_486, PMGC_2088, PMGC_2163, PMGC_2818 and PMGC_2879, from the *Populus* Molecular Genetics Cooperative (http://www.ornl.gov/sci/ipgc/ssr_resource.htm); and WPMS_14, WPMS_18 and WPMS_20 (Smulders *et al*. 2001).

Six standard measures of genetic diversity were assessed for each population: percentage of polymorphic loci (*P*), mean alleles per locus (*A*), effective number of alleles (*A*_e_), observed (*H*_o_) and expected (*H*_e_) heterozygosity and the fixation index over loci (*F*), as implemented by GenAlEx v6 (Peakall & Smouse 2006). The presence of null alleles was assessed using the program microchecker (van Oosterhout *et al*. 2004) as described by DeWoody *et al*. (2006). Evidence of a recent genetic bottleneck was assessed for each population using the Wilcoxon sign rank test for heterozygosity excess under the two-phased model of mutation as implemented by the program bottleneck (Luikart & Cornuet 1998; Luikart *et al*. 1998). Due to insufficient sample sizes, populations FR3, NE2 and SP1 were omitted from the microchecker analysis and populations FR3, FR4 and NE2 from the bottleneck analysis. Overall genetic differentiation was quantified as *Φ*_PT_ through the analysis of molecular variance (amova), and as individual locus *F*_ST_, as implemented by genalex v. 6 (Peakall & Smouse 2006).

Admixture was assessed using structure v2.3.3 (Pritchard *et al*. 2000; Falush *et al*. 2003). The likelihood of the observed data fitting a model of *K* genetic groups for the set *K* = {1:13} was estimated over five simulation runs, using a burn-in of 50 000 followed by 500 000 replications. Correlation in allele frequencies was allowed, and the remaining parameters were set to the default values. Two methods were used to infer the most likely value of *K*. First, the delta-K method of (Evanno *et al*. 2005) was applied using the Web-based tool structure harvester (Earl & vonHoldt 2012). Second, the distribution of the log-likelihoods was examined for the value with the highest probability and lowest variance.

To summarize the genetic differences over multilocus microsatellite genotypes, the pairwise genetic distances among individuals (Peakall *et al*. 1995; Smouse & Peakall 1999) were subjected to a principal coordinates analysis (PCoA) based on the covariance matrix with data standardization as implemented by genalex v6 (Peakall & Smouse 2006).

### Phenotypic measures: quantifying differences in morphology

In the spring of 2004, a common garden was established consisting of 512 genets planted as hardwood cuttings in six replicate, randomized blocks in a plantation located near the Institute of Forestry and Game Management near Geraardsbergen, Belgium (50.77N, 3.87E). Plantings were established on a grid with 0.75 × 2.0 m spacing, surrounded by a double row of the *Populus* cultivar ‘Muur’ to minimize edge effects. Once established, trees were cut back in early 2005 and regrowth pruned to a single dominant stem in June 2005. Site management, carried out by the local collaborators, included mechanical weed removal and fungicide application (Rohde *et al*. 2011). No irrigation or fertilizer was provided during the experiment. The number of replicates was reduced to three or four for the Spanish samples due to ramet mortality and labelling inconsistencies which were resolved using microsatellite data.

In the third year of growth (2006), each ramet of *P. nigra* in the common garden was examined for 12 morphological characteristics: five leaf traits, two biomass traits and five cell traits. Leaf measures included leaf area, leaf length, leaf width, leaf length:width ratio (LL_LW) and specific leaf area (SLA). Biomass traits were measured as stem height and circumference at the start of the 2006 growing season. Cellular traits were measured from epidermal imprints and included epidermal cell area (mm^2^), stomatal number, stomatal density (SD), stomatal index (SI) and cell number per leaf (CN). Detailed methods are provided in the Methods S1 (Supporting information).

To explore the variation in individual morphological traits, differences among population means were tested for each trait (*y*) using the model *y* = *a*_*x*_ + *b*_*y*_ + *ɛ*, where *a*_*x*_ is the population mean, *b*_*y*_ is the genotype nested within each population, and *ε* is the error. Scores for LL_LW were arcsine-transformed, and SD were log-transformed prior to analyses. This linear mixed model was implemented over genotype means using the lme function provided in the nlme package in r (R Project for Statistical Computing, Inc.) In addition, phenotypic correlations were estimated for each pair of traits using spss (v. 15.0.0; SPSS, Inc., Chicago, IL, USA).

To assess overall morphological differentiation of *P. nigra* populations for use in the IBA analyses, a discriminant analysis was performed on the genotype means of each trait measure. Those factors significantly contributing to differences between populations were used to visualize overall morphological differences between individuals and populations. Discriminant analyses were implemented using the Factor Analysis and Classification Analysis functions in spss (v. 15.0.0; SPSS, Inc.).

### Tests for IBD and IBA

Four matrices were built to describe differences between populations of *P. nigra*: (i) genetic differences calculated as the Euclidian distance between the population means for the first two coordinates of the PCoA, (ii) morphological differences assessed as the Euclidian distance between the population means for the first two factors of the discriminant classification analysis, (iii) climate differences calculated as the Euclidian distance between the population means for the two climatic variables from the PCA and (iv) geographic distance from the latitude and longitude of the site of sample collection. To assess IBD, IBA and infer IBC, each response matrix (genetic and morphological differences) were compared to the two predictor matrices (climate differences and geographic distance) using simple Mantel tests (Smouse *et al*. 1986; Manly 1997). The simple Mantel tests were performed in genalex v. 6 (Peakall & Smouse 2006), with significance determined via permutation tests.

To test for IBA directly, correspondence between pairwise genetic, morphological and geographic distances was assessed using partial Mantel tests The first tested for IBD as the correlation between genetic and geographic differences when controlling for morphological differences (Gen, Geo ¦ Morpho). The second tested for IBA as the correlation between morphological and genetic differences when controlling for geographic distances (Gen, Morpho ¦ Geo). Partial Mantel tests were implemented using the Isolation by Distance Web Service v. 3.15 (Jensen *et al*. 2005), with significance determined via permutation tests. Where significant IBA was observed, regression analysis was used to describe the relationship of the residual morphological values vs. the residual genetic values (the partial regression plots) for all pairs of populations, comparisons of small-leaf to small-leaf populations, large-leaf to large-leaf populations, and small-leaf to large-leaf populations (Moya-Larano & Corcobado 2008), with significance determined using anova. All regression analyses were performed in spss v17 (SPSS, Inc.).

## Results

### Climate differences among sampling sites

The eight climate variables and day length varied across the sampling sites (Table 2). The principal components analysis revealed distinct climates based on the minimum temperature of the coldest month (MinTemp), the seasonality of temperature (SDTemp), mean precipitation of the wettest month (MaxPpt), and the maximum day length (MaxDay) (Fig.1). The first two components accounted for 84% of the variance in the data. These measures distinguished the sites in Spain, in Italy and in the Netherlands, with sites in France and Germany intermediate.

**Table 2:** Climatic variables at 13 sites sampled for *Populus nigra*

Pop.	MAPpt	VarPpt	MaxPpt	MinPpt	MATemp	SDTemp	MaxTemp	MinTemp	MaxDay
FR1	890	15	83	48	10.3	6259	25.7	−2.4	15.6
FR2	840	20	95	41	12.4	6357	28.1	0	15.6
FR3	688	26.4	85	23.6	12.8	5945	27.4	0.6	15.5
FR4	707	11.3	72	47.7	11	5708	24.8	−0.1	16
FR5	711	17.5	77	44.3	11.6	5145	24.4	1.6	16
GE	590	19	65	36.1	9.9	6525	24.5	−1.8	16.3
IT1	982	23	122	55	13	7248	29	−1	15.7
IT2	966	23	121	55	13	7296	29	−0.9	15.7
NE1	774	15.3	77	47.3	9.3	5451	21.4	−0.7	16.8
NE2	802	14.8	81	50.3	9.7	5359	21.1	0.2	16.5
NE3	791	14.3	77	46.9	9.5	5383	21.4	−0.3	16.7
SP1	439	26	56	20	14.1	6097	29.7	1.8	15.2
SP2	365	31	53	17	13.7	6243	29.5	1.3	15.2

MAPpt, mean annual precipitation (mm); VarPpt, precipitation seasonality; MaxPpt, mean precipitation of the wettest month (mm); MinPpt, mean precipitation of the driest month (mm); MATemp, mean annual temperature (°C); SDTemp, seasonality of temperature; MaxTemp, maximum temperature of the warmest month (°C); MinTemp, minimum temperature of the coldest month (°C); MaxDay, maximum day length (h).

### Patterns of neutral genetic structure in *Populus nigra*

Microsatellite variation revealed high levels of polymorphism and moderate levels of allelic diversity and heterozygosity, with no evidence of allele fixation (Table 3). Null alleles were detected in three populations at locus PMGC_2088 (IT1, freq. 0.09; NE1, freq. 0.11, NE3, freq. 0.27) and at low frequency in a single population at WPMS_14 (FR5, freq. 0.15) and WPMS_20 (IT1, freq. 0.09). Low rates of mismatches between repeated samples resulted in low error rates: 3.3% per allele or 4.6% per reaction.

**Table 3:** Genetic diversity (mean number of samples, mean alleles per locus, effective alleles per locus, observed and expected heterozygosity, and fixation) observed at nine microsatellite loci in 13 populations of *Populus nigra*

Population	*N*	*A*	*A*_e_	*H*_o_	*H*_e_	*F*
FR1	30.2	9.2	4.9	0.774	0.770	−0.006
FR2	42.4	11.2	6.0	0.799	0.808	0.012
FR3	5.9	5.4	4.0	0.727	0.708	−0.057
FR4	6.0	5.9	4.2	0.796	0.736	−0.084
FR5	12.1	7.0	5.0	0.755	0.751	−0.012
GE	42.0	9.0	4.3	0.767	0.743	−0.026
IT1	39.0	10.7	5.6	0.787	0.797	0.019
IT2	30.4	9.6	6.1	0.817	0.802	−0.021
NE1	22.4	8.2	4.8	0.813	0.777	−0.049
NE2	5.0	5.2	4.2	0.867	0.749	−0.168
NE3	8.7	5.0	3.5	0.735	0.693	−0.054
SP1	15.3	6.1	4.2	0.640	0.719	0.071
SP2	27.6	8.4	4.8	0.734	0.738	0.005
Overall	22.1	7.8	4.7	0.770	0.753	−0.029

Sign tests for heterozygosity excess at Hardy–Weinberg equilibrium compared to that expected at mutation–drift equilibrium was observed for populations IT1 (*P* < 0.01), NE3 and SP1 (*P* < 0.05), indicating these populations have undergone a recent population bottleneck. Results for populations NE3 and SP1 may be influenced by the small sample size in this analysis. The bottleneck in population IT1 may have contributed to increased inbreeding and homozygosity identified as possible null alleles (above).

Overall genetic differentiation was significant (*Φ*_PT_ = 0.120, *P* < 0.001), indicating gene flow among populations of *P. nigra* is restricted across western Europe. Per-locus estimates ranged from 0.044 to 0.118 (mean = 0.072, standard deviation = 0.021) (Supporting Information).

**Fig. 2:**
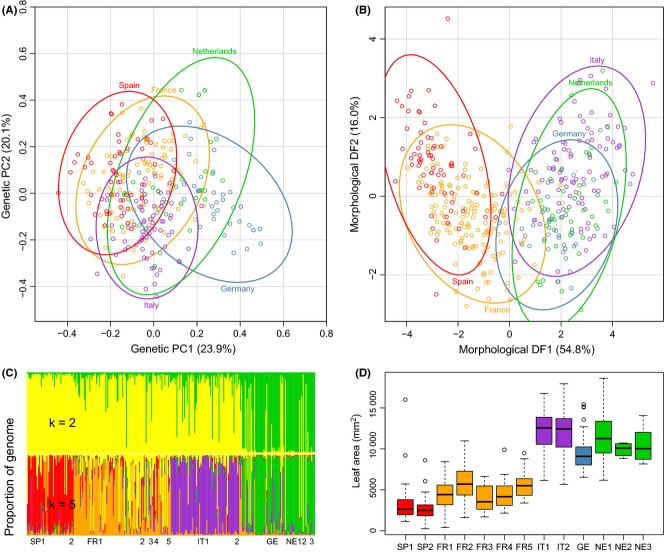
(A) Principal coordinates analysis of microsatellite data reveals patterns of differentiation among populations of *Populus nigra*. (B) Discriminant classification analysis of 12 morphological traits reveals significant geographic structuring to phenotypic variation in *Populus nigra*. Each point represents a single genet, with colour corresponding to country of origin. Ellipses represent the 95% confidence intervals for the population means. Population means for each data type were used for pairwise comparisons in full and partial Mantel tests. (C) Admixture analysis of microsatellite data identified two (top) or five (bottom) genetic groups. Assignment of individual trees (vertical bars) roughly corresponded to geographic origin, with samples from France displaying greater admixture. Position of abbreviations below indicates the order and extent of each population. (D) Morphological differentiation among populations is typified by variation in leaf area quantified in a common garden study. Each box-and-whisker plot represents the observed measures for each population, with the centre bar indicating the median value.

The number of genetic clusters (*K*) identified by the admixture analyses varied with the method of inference used. The structure harvester method of Evanno *et al*. (2005) identified *K* = 2 as most likely. Individual assignment tests over two genetic groups indicated the collections from Spain, France and Italy were distinct from trees from Germany and the Netherlands (Fig.2C). As experience indicates the Evanno method may be overly conservative in species with significant gene flow, to identify the mostly likely biologically meaningful value of *K*, direct examination of likelihood ratios indicated the tests for *K* = 5 genetic clusters resulted in the highest likelihood without an increase in variance (Fig. S1, Supporting information). Individual assignment tests to five genetic groups roughly correspond to country of origin (Fig.2C), with the samples from Germany and the Netherlands assigned to the same genetic cluster and samples from France displaying the greatest admixture.

The PCoA revealed overall differences to be of a smaller magnitude among individual trees than populations (Fig.2A). The first two components explained a cumulative total of 44.0% of the variance among individuals. Significant overlap was observed between populations.

### Phenotypic differentiation among populations

All measures of phenotype varied significantly among populations (Table 4). Several traits displayed bimodal distributions across the collection. For instance, leaf size was strongly bimodal, with samples from Spain and France having smaller leaves than those from Italy, Germany or the Netherlands (Fig.2D, Tables S1–S5, Supporting information).

**Table 4:** Morphological variation in leaf, cell and biomass traits measured in a common garden study of *Populus nigra* from 13 natural stands in western Europe revealed significant phenotypic variation. Discriminant factor analyses were used to reduce the multivariate data into two components for isolation by adaptation comparisons

Trait	*F*-ratio	*P*-value	Component 1	Component 2
Leaf area	*F*_12,499_ = 135.1	<0.0001	0.948	−0.179
Leaf length	*F*_12,499_ = 133.7	<0.0001	0.935	−0.178
Leaf width	*F*_12,499_ = 111.4	<0.0001	0.938	−0.218
Leaf length:width	*F*_12,493_ = 3.265	<0.0001	0.931	−0.168
Specific leaf area	*F*_12,467_ = 160.0	<0.0001	0.520	0.697
Cell area	*F*_12,453_ = 3.36	<0.0001	0.487	0.742
Cell number per leaf	*F*_12,451_ = 67.8	<0.0001	0.248	0.660
Number of stomata (abaxial)	*F*_12,498_ = 10.5	<0.0001	0.923	0.044
Stomatal density (abaxial)	*F*_12,489_ = 6.27	<0.0001	0.832	−0.205
Stomatal index (abaxial)	*F*_12,443_ = 2.659	0.0019	0.878	−0.198
Height (second year)	*F*_12,449_ = 44.7	<0.0001	−0.288	0.108
Circumference (third year)	*F*_12,499_ = 78.4	<0.0001	−0.307	−0.467
% variation			54.8	16.0

Correlations between morphological measures were highly significant but varied within and among trait classes (leaf traits, cell traits and biomass traits; Supporting information). The discriminant factor analysis (DFA) was required to reduce the variation of the highly correlated phenotypic measures to a smaller number of orthogonal factors (Table 4). The first two components of the DFA described cumulative total of 71% of the variation and revealed significant geographic patterning in morphology. Samples from Spain were morphologically distinct from those from Italy, Germany and the Netherlands, with the French populations intermediate (Fig.2B). Leaf area was correlated with the first factor (Table 4), indicating that identifying collections as small leaf (Spain and France) or large leaf (Germany, Italy and the Netherlands) may represent the two general morphotypes (Viger 2011).

### Tests for IBD, by colonization and by adaptation

Simple Mantel test identified significant correlations between the genetic, morphological, geographic and climatic matrices distance matrices (Table 5). Correlations were strongest between the morphological and geographic matrices (*r* = 0.582, *P* < 0.001), genetic and geographic matrices (*r* = 0.577, *P* < 0.001) and morphological and genetic matrices (*r* = 0.522, *P* < 0.001). Weaker but significant correlations were observed between the morphological and climate matrices (*r* = 0.406, *P* < 0.001) and the genetic and climate matrices (*r* = 0.399, *P* = 0.004).

**Table 5:** Correlations between genetic (from PCoA), morphological (from discriminant factor analysis), climatic (from principal component analysis) and geographic (km) differences among 13 populations of *Populus nigra* tested with simple and partial Mantel tests

Comparison	*r*	*P*-value
Simple Mantel tests		
Genetic, Geographic	0.633	<0.001
Genetic, Climate	0.551	<0.001
Morphological, Geographic	0.582	<0.001
Morphological, Climate	0.416	0.002
Morphological, Genetic	0.622	<0.001
Partial Mantel tests		
Genetic, Geographic | Morphological	0.426	<0.001
Genetic, Climate | Morphological	0.418	0.004
Morphological | Genetic, Geographic	0.403	0.004
Morphological | Genetic, Climate	0.522	<0.001

Significant correlation between genetic and geographic distance, independent of morphological divergence (Gen, Geo¦Morpho), indicates that IBD has influenced the genetic structure of *P. nigra* in western Europe (Table 5). Genetic differences increased as a function of distance, consistent with the genetic pattern resolved by the admixture analyses. Tests using pairwise *F*_ST_ (Rousset 1997) measures and log-transformed data were significant and concordant (Tables S1–S5, Supporting information).

Isolation by adaptation also contributed to the genetic structure of populations, as genetic similarity increased with phenotypic similarity, even when controlling for geographic structure among populations (Gen, Morpho¦Geo). Analyses of log-transformed data were consistent (data not presented). This result indicates that genetic differentiation at microsatellite loci may be influenced by reduced gene flow between morphologically distinct populations or reflect historic vicariance due to IBC.

Examination of partial regression plots of the genetic and morphological differences between populations further revealed the putative source of IBA (Fig. 4). IBA was significant among comparisons of all pairs of populations (*F*_1,76_ = 28.1, *P* < 0.001; slope = 0.038, 95% CI 0.023–0.052). Categorical comparisons of small-leaf and large-leaf pairs revealed the trend to be driven by differences among the small-leaf populations. Significant positive relationships were observed in small-leaf to small-leaf comparisons (*F*_1,19_ = 17.7, *P* < 0.001; slope = 0.07, 95% CI 0.035–0.105), and small-leaf to large-leaf comparisons (*F*_1,40_ = 26.5, *P* < 0.001; slope = 0.053, 95% CI 0.032–0.074), but not large-leaf to large-leaf comparisons (*F*_1,13_ = 3.52, *P* = 0.083; slope = 0.02, 95% CI −0.003–0.043). Thus, the genetic and morphological variation observed in the large-leaf populations was fully explained by IBD, with gene flow decreasing as a function of distance. However, when comparing small-leaf to small-leaf, or small-leaf to large-leaf populations, the genetic differentiation between populations resulted from IBA as well, and populations are more genetically dissimilar than their geographic distance would predict.

## Discussion

In the strictest terms, evidence of local adaptation requires that a species displays multiple morphotypes, each having higher fitness in its native habitat than the others, as confirmed through reciprocal transplant experiments (Kawecki & Evert 2004; Savolainen *et al*. 2013). Adaptive differentiation, however, can be described by examining patterns of genetic differences and climatic variation (Sork *et al*. 2010; Salmela 2014). Our study of *P. nigra* revealed distinct morphological and genetic variation in trees from across western Europe and related these patterns to differences in local climate at each site. We conclude that adaptive differentiation and persistent IBC acted in combination to produce the observed genetic and morphological patterns.

**Fig. 3:**
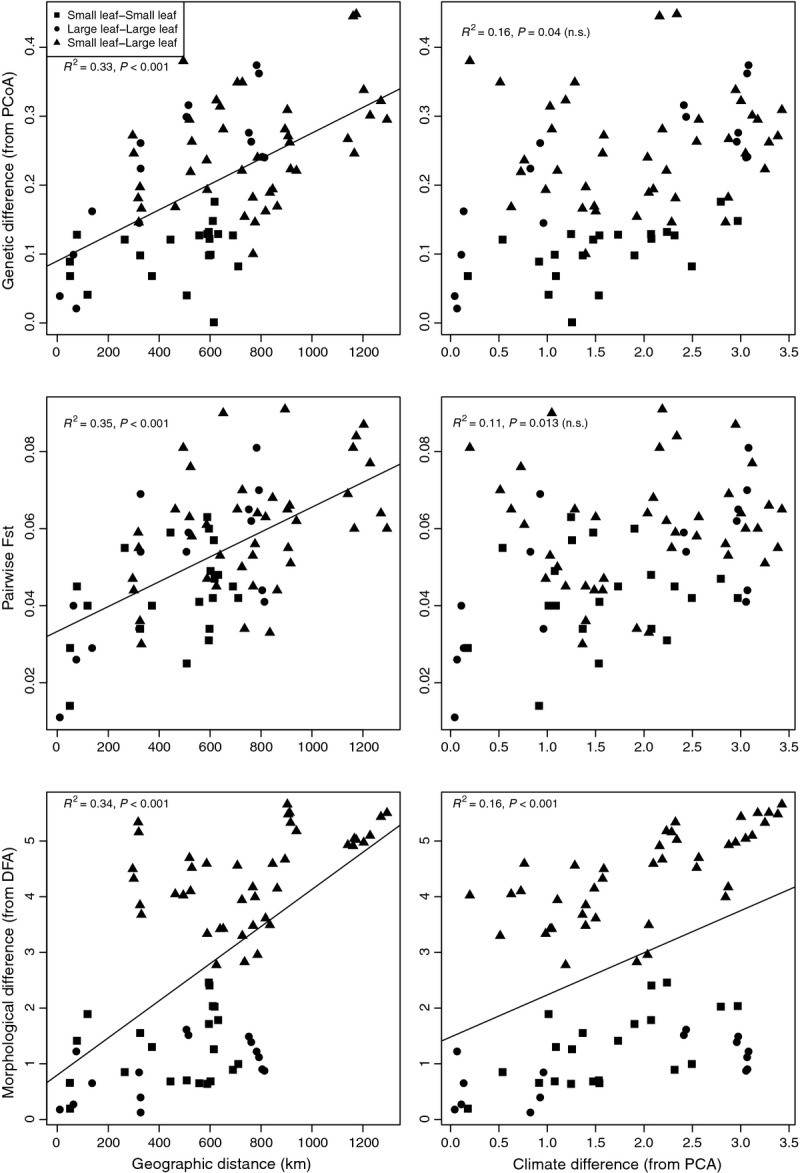
Genetic differences between populations (from PCoA, top row), allele frequency variance (*F*_ST_, middle row) and morphological differences between populations (from discriminant factor analysis, bottom row) all correlate with geographic distance (left panels) more than climatic differences (right panels) between sampling sites. Note that high levels of morphological differences are observed between sites with similar climates (triangle symbols, bottom right pane). Correlations from simple Mantel tests, with significance determined using permutation tests. n.s. = *P*-values are nonsignificant after sequential Bonferroni corrections.

Leaf size, branching architecture and growth rate are all considered adaptive traits in trees and, in particular, may be linked to water availability (Dudley 1996a,b; Picotte *et al*. 2007; Yang *et al*. 2014). Species or morphotypes adapted to lower or seasonal precipitation tend to display smaller, thicker leaves, greater branching and slower growth rate in response to the environmental stress (Poorter *et al*. 2009; De Kort *et al*. 2014). In our common garden experiment, trees from Spain and France displayed small leaves, a branching architecture and smaller circumference, while trees from northern Italy, Germany and the Netherlands displayed large leaves, a straight architecture and large circumference. If these morphotypes are strictly adaptive, we would expect small-leaf trees to inhabit similar environments, and those would be distinct from climates experienced by large-leaf trees. Although the correlation between morphological difference and climatic difference was significant in *P. nigra* (Fig.3), the pattern did not reveal that different morphotypes inhabit different environments. Examination of nine climatic variables revealed the sampling locations to have diverse climates, with some climates in France (small-leaf populations) similar to the site in Germany (large-leaf), although climates in Spain (small-leaf), Italy (large-leaf) and the Netherlands (large-leaf) were all distinct (Fig.1).

**Fig. 4:**
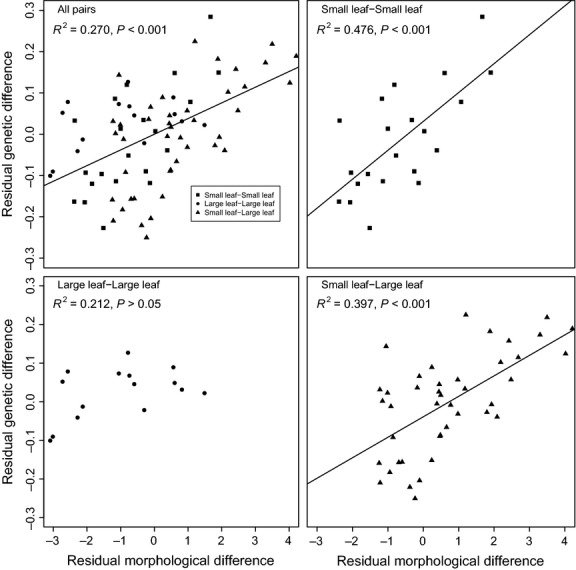
Partial regression plots illustrating correlated genetic and morphological differences between populations of *Populus nigra* after controlling for the effects of geographic distance. Points represent pairwise comparisons between two small-leaf populations (squares), two large-leaf populations (circles) or populations with differing morphology (triangles). Lines represent significant regressions of the residuals, with significance determined using anova. Comparisons of large-leaf to large-leaf populations revealed no significant relationship. Significance did not change after Bonferroni corrections.

Further, if selection was strong enough to reduce successful gene flow between morphotypes, we would expect residuals from the partial correlations (i.e. IBA; Nosil *et al*. 2009), to be driven by comparisons of different morphotypes (e.g. small-leaf to large-leaf). The significant residual correlation in *P. nigra* was observed in small-leaf to large-leaf comparisons, but also in small-leaf to small-leaf comparisons, indicating that variation within the small-leaf morphotype in particular may be adaptive (Fig.4). For comparison, in a similar study of adaptive differentiation in *Alnus*, correlation between phenotypic and neutral genetic structure was significant but weak (_adj_*R*^2^_cum_ = 0.05, *P* < 0.001), and no evidence was observed for IBD (De Kort *et al*. 2014).

Isolation by distance and IBC due to serial recolonization have also contributed to the morphological and genetic structure of *P. nigra* and may explain the inconsistency between morphology and climate in the populations from France. Several tree species maintain signatures of postglacial range expansions in their extant genetic structures (Vendramin *et al*. 2000; Petit *et al*. 2002; Palme *et al*. 2003; Tzedakis *et al*. 2013), although local adaptation is expected to remove these patterns from morphological traits (Cavers *et al*. 2004). For species existing as a metapopulation (*P. nigra* better fits a model of isolated populations requiring regular disturbance than the canonical climax forest tree), current genetic structure may reflect historic rather than extant demographic patterns (Orsini *et al*. 2008), although differences between IBD and IBC are difficult to distinguish using neutral markers (Orsini *et al*. 2013). If gene flow is insufficient to overcome the historic differentiation, a signature of historic vicariance may persist in the morphological differences between populations. The strongest evidence of IBC would come from comparing differences at slow-evolving or maternally inherited loci (e.g. chloroplast haplotypes), morphological differences and geographic distance. As the sole process influencing a species, IBC is expected to result in a lack of correlation between genetic differentiation and distance (IBD) or environment (IBA; Orsini *et al*. 2013). Although our study lacks direct tests of postglacial migration from plastid sequences, the pattern of postglacial recolonization in *P. nigra* has been previously described. Cottrell *et al*. (2005) identified up to three putative refugia and demonstrated that trees in France displayed haplotypes more similar to Iberian populations than those from central Europe. These patterns of plastid variation are generally similar to the pattern of morphological variation observed in the common garden study, where small-leaf trees from Spain and France overlapped in morphology, but were generally distinct from large-leaf trees from central Europe. There are three possible explanations for the similarities in the plastid and morphological patterns: (i) the plastid variation reflects adaptive differences and thus IBA, (ii) the morphological differences maintain a residual signature of the historic vicariance from the last glacial maximum, and (iii) the patterns are similar due to the influence of a third untested process. Given the growing literature demonstrating that plastid genomes are not strictly neutral (Bock *et al*. 2014), the congruence between the morphological differences and signature of recolonization described by plastid variation (Cottrell *et al*. 2005) may be adaptive. As with correlations between morphology and climate, if the plastid (historic) variation mirrors the selective differentiation, we would expect plants with similar morphology to inhabit similar environments, yet the diversity of climates inhabited by small-leaf populations in France (Fig.1) is inconsistent with this expectation. The morphological variation in *P. nigra* is consistent with the patterns of postglacial recolonization reported in a number of tree species, indicating morphology may reflect, in part, IBD due to serial colonization. However, we cannot rule out the third possibility that we have failed to identify the causative process underlying these correlations. Historic and extant population structure is gaining recognition as a potential force shaping morphological variation in forest trees.

Efforts to correlate phenotypic variation and specific climatic variables have met with mixed success. Phenological traits tend to vary with latitude or maximum day length of the origin site, consistent with strong selective pressure from growing season length on tree species (Hall *et al*. 2007; Rohde *et al*. 2011; De Kort *et al*. 2014; McKown *et al*. 2014). Evidence of phenotypic variation, especially biomass or ecophysiological traits, correlating with climatic variables related to precipitation or temperature is less common, but has been reported (Kelly *et al*. 2003; Royer *et al*. 2008; De Kort *et al*. 2014). Overall, these association studies demonstrate the difficulty of linking morphological variation to a single correlating (putatively causative) climate variable. Our multivariate approach allows a landscape-level comparison of population differences, providing evidence of adaptive differentiation at a broad scale (Sork *et al*. 2010; McKown *et al*. 2014).

The use of structure to infer the number of genetic clusters in a collection is an ad hoc application of the software commonly applied in population genetic studies (Pritchard *et al*. 2000). Multiple methods have been proposed to interpret the structure results, yet those methods often appear overly conservative (Earl & vonHoldt 2012) or liberal (Falush *et al*. 2003) in assigning the number of genetic clusters. Here, the Earl & vonHoldt (2012) method indicated *K* = 2, with the Italian samples clustering with those from France and Spain. This pattern varies from those reported in previous studies, where populations from Italy are more similar to the central European trees, and is likely a consequence of the sampling design and limited number of microsatellite data. The Italian trees formed a distinct cluster at *K* = 3 (data not presented). Attempting to identify biologically meaningful differences, we considered the *k* of the highest probability maintaining a small variance, *K* = 5, in which samples clustered according to country of origin, except those from Germany and the Netherlands clustered together. Samples from France were admixed between two genetic clusters. This pattern of admixture and differentiation was more consistent with previous AFLP and microsatellite data (Smulders *et al*. 2008).

In addition, anthropogenic factors may have contributed to the extant genetic structure of *P. nigra*. Tests of genetic bottlenecks identified three populations that may have recently undergone a population bottleneck. The heterozygosity excess indicated in three populations refers to the levels expected a mutation–drift equilibrium (not Hardy–Weinberg equilibrium), as estimated by the number of alleles in each sample. Human actions may have particularly affected population IT1, sampled along the Ticino River. A previous study reported the depletion of the natural poplar stands in the Ticino River region in northern Italy during the Second World War (Fossati *et al*. 2003), likely contributing to the genetic bottleneck identified from the microsatellite data and demonstrating the potential for management events to affect standing genetic variation in forest trees.

Understanding how IBD, IBA and IBC individually and jointly affect the genetic structure of a keystone species is critical for conservation and management, especially in context of a changing climate. Numerous models predicting climate changes for the next century have been published, and while results are variable (Blenkinsop & Fowler 2007), some consistent trends have emerged. In Europe, the mean temperature is expected to increase, following a large cline across latitudes, with the greatest increases in the northern areas (Raible *et al*. 2006; Gessler *et al*. 2007). In addition, rates and distribution of precipitation are also expected to change. Models have predicted that summer precipitation will decrease over much of Europe, with levels increasing only in the most northern latitudes (Blenkinsop & Fowler 2007). Drought events are predicted to increase in frequency (Blenkinsop & Fowler 2007; Penuelas *et al*. 2007), especially in southern, Mediterranean areas (Blenkinsop & Fowler 2007). These changes in temperature and precipitation will change the distribution of climate types across Europe. The distribution and abundance of climates analogous to those observed in 1945 (i.e. prewarming) are predicted to decrease in size, increase in fragmentation and generally shift to the northeast, with novel climatic conditions developing (Ohlemuller *et al*. 2006).

If morphological differentiation strictly tracks differences in environment, then comparing the current distribution of morphotypes and predicted distribution of environments may provide insight for conservation efforts (Sork *et al*. 2010; Kremer *et al*. 2012). However, if the morphological differentiation reflects a combination of adaptive and nonadaptive processes (such as IBC), predicting species response to climate change is further complicated, as the standing genetic variation may not match the most adaptive phenotype for an environment. For example, the *P. nigra* populations from central France (FR4 and FR5) experience a climate more similar to the site in Germany than Spain, but display morphology more similar to the Iberian collections (Fig.1). If the small-leaf morphotype is adapted to lower or more seasonal precipitation due to their Iberian ancestry (IBC), rather than the current climate, these populations may be ‘pre-adapted’ to higher drought frequency over the next century (Hu & He 2006; Kremer *et al*. 2012). Further, we predict the admixed nature of the populations in France may result in greater plasticity in response to varying environments. An ongoing study of two common garden experiments in contrasting environments (England and Italy) is expected to provide additional insight into the genetic basis of phenotypic differentiation in this important forest tree.

Together, these patterns of morphological, genetic, geographic and climate differences indicate that IBC due to serial colonization events likely continues to influence the morphological and genetic structure in a forest tree. Genetic structure was significantly correlated with geographic distance but not climatic differences, consistent with models of IBD or isolation by serial colonization. Morphological variation was correlated with both geographic distance and climatic differences. These patterns confirm that multiple evolutionary processes influence the morphological and genetic structure of long-lived species and indicate that the response to climate change may be influenced by historic factors.

## Data accessibility

Morphological measures, microsatellite data and climate details are available in the Dryad Digital Repository under doi: 10.5061/dryad.kq0n5.

## Supporting information

**Fig. S1** Results of the structure and structure harvester analyses of microsatellite data.

**Methods S1** Details of microsatellite analyses and morphological data collection, with associated references.

**Tables S1–S5** Correlation among climatic variables; Per-locus *F*_ST_ values; Phenotypic variation measured at 12 traits in trees from 13 populations grown in a common garden study; Correlation among population means for 12 morphological traits.
