# Supplementary material for: Genetic and morphological differentiation in Populus nigra L.: isolation by colonization or isolation by adaptation?
Source: Mol Ecol. 2015 May 14;24(11):2641–55. doi: 10.1111/mec.13192 (PMC4692097; doi:10.1111/mec.13192)
Supplement: Supplementary file 1 — Fig. S1 Results of the structure and structure harvester analyses of microsatellite data. [file mec0024-2641-sd1.pdf]

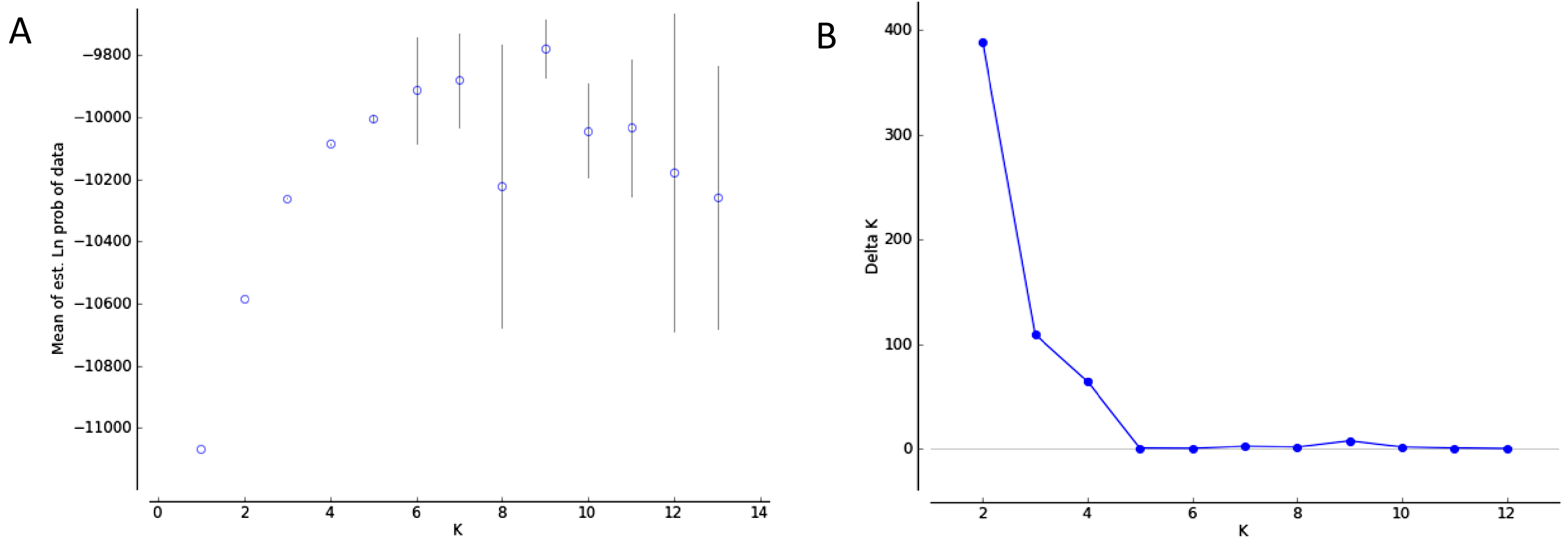

Supplementary Figure. Admixutre analyses identify a small number of genetic groups in the collection of *P. nigra* from 13 geographic populations. (A) The observed likelihood values for the set of K={1:13} genetic groups tested using MCMC simulations. The value where the rate of increase in likelihood diminishes without an increase in variance corresponds to K=5. (B) The delta-K method of Evanno et al. (2005) indicates two genetic groups to be present.
