# Supplementary material for: Genetic and morphological differentiation in Populus nigra L.: isolation by colonization or isolation by adaptation?
Source: Mol Ecol. 2015 May 14;24(11):2641–55. doi: 10.1111/mec.13192 (PMC4692097; doi:10.1111/mec.13192)
Supplement: Supplementary file 2 — Methods S1 Details of microsatellite analyses and morphological data collection, with associated references. [file mec0024-2641-sd2.pdf]

Microsatellite analyses

Leaves from each genet in the common garden experiment were sampled, flash frozen in liquid nitrogen and stored at -80°C until processed. Total genomic DNA was isolated from each sample using the Qiagen® DNeasy Mini kit (Crawley, UK) following the manufacturer's instructions. The quality and quantity of each sample was assessed using a ThermoScientific® NanoDrop 1000 spectrophotometer (Wilmington, Delaware, USA) following the manufacturer's instructions.

A panel of nine bi-parentally inherited, co-dominant microsatellite markers was assayed for each sample. Primer sequences for each locus were obtained from the literature (PMGC_14, PMGC_486, PMGC_2088, PMGC_2163, PMGC_2828, and PMGC_2879 from the *Populus* Molecular Genetics Cooperative (http://www.ornl.gov/sci/ipgc/ssr_resource.htm); WPMS_14, WPMS_18 and WPMS_20 from (Smulders *et al.* 2001). The forward primer of each pair was modified to incorporate a standard M13 sequence allowing incorporation of a fluorescent M13 primer during amplification (Schuelke 2000). In addition, the reverse primer was modified with the 3' addition of 5'-GTTTCTT-3' in order to prevent the non-template addition of dATP by *Taq* polymerase.

Each locus was amplified in a separate polymerase chain reaction (PCR®) containing 1X reaction buffer (supplied with enzyme), 1.5 mM $MgCl_2$, 2 nmoles of each dNTP, 0.5 pmole of the unlabelled locus-specific forward primer, 5 pmole of the locus-specific reverse primer, 5 pmole of the fluorescently-labelled M13 primer, and 0.2 Units Qiagen HotStarTaq® DNA polymerase in a 10.0 µL total reaction volume. Amplifications conditions included a hot start of 95°C for 15 min., followed by 42 cycles of 94°C for 20 sec., 55°C for 30 sec., and 72°C for 30 sec., with a final extension at 72°C for 5 min. Locus GCPM_2180-1 required 2.0 mM $MgCl_2$ for amplification. In order to assess amplification quality, ten samples (16%) were replicated for each locus. Capillary electrophoresis was carried out on an ABI3730 (Applied Biosystems, Inc., Foster City, California, USA) systems by GeneService Limited (Nottingham, UK). Results were analyzed and scores assigned using Peak Scanner v. 1.0 (Applied Biosystems, Inc., Foster City, California, USA).

Morphological measures

In the third year of growth (2006), each ramet of Populus nigra in the common garden for was examined for 12 morphological characteristics: five leaf traits, two biomass traits, and five cell traits. For each ramet, the youngest fully mature leaf on the leader stem was collected and its image scanned at 200 DPI using an Umax Astra 6700 scanner. Digital images were assessed using the analysis software Image J (Image J.1.32j, Wayne Rasband, USA) to calculate the leaf area, leaf length from tip to petiole, and leaf width at the widest point. The ratio of leaf length to width was calculated from these measurements. In the second year of growth (2005) one mature leaf was oven dried and the specific leaf area (SLA) then calculated as the ratio of leaf area (mm2) to dry mass (mg). To estimate biomass, stem height and diameter were measured at the beginning of the 2006 growing season.

Cellular imprints were taken from the abaxial surface of a single mature leaf from each ramet by covering an approximately 1 cm2 area on the basal section of the leaf following (Ferris et al. 2002). Cell imprints were captured as digital images using a Zeiss microscope at 400X magnification. Images were processed in Image J. For each field of view, the number of epidermal cells and number of stomata were counted, and the average cell area (mm2) calculated from the areas of 10 randomly chosen epidermal cells. From these data three measures were calculated: stomatal density (SD) as number of stomata per field of view; stomatal index (SI) of the abaxial surface as the ratio of stomata to the total number of cells per field of view (x100), and the number of cells per leaf (CN) as the ratio of leaf area (mm2) to epidermal cell area (mm2).

References

Ferris R, Long L, Bunn SM*, et al.* (2002) Leaf stomatal and epidermal cell development: identification of putative quantitative trait loci in relation to elevated carbon dioxide concentration in poplar. *Tree Physiology* **22**, 633-640.

Schuelke M (2000) An economic method for the fluorescent labeling of PCR fragments. *Nature Biochemistry* **18**, 233-234.

Smulders MJM, van der Schoot J, Arens P, Vosman B (2001) Trinucleotide repeat microsatellite markers for black poplar (Populus nigra L.). *Molecular Ecology Notes* **1**, 188-190.
