# Supplementary material for: Genetic and morphological differentiation in Populus nigra L.: isolation by colonization or isolation by adaptation?
Source: Mol Ecol. 2015 May 14;24(11):2641–55. doi: 10.1111/mec.13192 (PMC4692097; doi:10.1111/mec.13192)
Supplement: Supplementary file 3 — Tables S1–S5 Correlation among climatic variables; Per-locus FST values; Phenotypic variation measured at 12 traits in trees from 13 populations grown in a common garden study; Correlation among population means for 12 morphological traits. [file mec0024-2641-sd3.pdf]

Pairwise correlations among climate variables at 14 sites sampled for *P. nigra*. Significance determined using one-tailed tests.

	MAPpt	VarPpt	MaxPpt	MinPpt	MATemp	SDTemp	MaxTemp	MinTemp	MaxDay
**MAPpt**	---	-0.462*	0.878***	0.881***	-0.291	0.270	-0.185	-0.604*	0.298
**VarPpt**		---	-0.028	-0.716**	0.803***	0.479*	0.745**	0.429	-0.653**
**MaxPpt**			---	0.665**	0.131	0.582*	0.205	-0.411	0.003
**MinPpt**				---	-0.503*	0.074	-0.416	-0.584*	0.513*
**MATemp**					---	0.524*	0.939***	0.515*	-0.885***
**SDTemp**						--	0.724**	-0.405	-0.490*
**MaxTemp**							--	0.217	-0.925***
**MinTemp**								---	-0.307
**MaxDay**									---

*** P<0.001
** P<0.01
* P<0.05

Per-locus F_{ST} estimates for nine microsatellite loci examined in 13 populations of *Populus nigra*.

Locus	F_{ST}
PMGC_14	0.075
PMGC_486	0.062
PMGC_2088	0.088
PMGC_2163	0.063
PMGC_2818	0.069
PMGC_2879	0.044
WPMS_14	0.072
WPMS_18	0.058
WPMS_20	0.118

Population means (standard errors)

Population	N	LA	LL	LW	LL_LW	SLA	Stomata	SD	SI	CA	CNPL	Height	Diameter
FR1	61.9	4474.6 (202.8)	79.5 (1.9)	71.8 (1.9)	37.7 (4.9)	1081.3 (54.4)	22.4 (0.443)	158.4 (4.4)	7.674 (0.251)	6.430 (0.054)	1.451 (0.084)	188.8 (5.07)	11.1 (0.48)
FR2	61.8	5827.5 (253.2)	87.9 (1.8)	85.9 (2.3)	32.7 (6.9)	1323.7 (72.7)	23.8 (0.446)	167.7 (5.0)	8.362 (0.252)	6.250 (0.053)	2.123 (0.118)	214.9 (5.53)	14.8 (0.60)
FR3	11.5	3905.4 (478.4)	71.0 (3.8)	67.3 (3.6)	17.8 (4.7)	554.5 (129.1)	24.0 (1.731)	174.0 (16.6)	8.834 (0.680)	6.315 (0.150)	1.420 (0.337)	157.0 (12.37)	7.2 (1.22)
FR4	24.6	4434.3 (341.8)	73.9 (2.5)	73.2 (3.2)	28.9 (6.9)	864.7 (91.0)	24.9 (0.782)	228.6 (29.4)	8.367 (0.513)	6.457 (0.153)	1.585 (0.290)	185.6 (9.37)	9.1 (0.78)
FR5	20.6	5709.7 (373.4)	83.4 (2.5)	84.4 (3.1)	21.3 (6.4)	1240.3 (136.0)	23.2 (0.840)	180.8 (9.1)	8.158 (0.363)	6.288 (0.118)	1.985 (0.199)	203.0 (11.16)	12.5 (1.20)
GE	53.8	9405.4 (264.9)	112.7 (1.7)	107.0 (1.4)	20.8 (3.4)	2539.4 (64.1)	26.8 (0.602)	196.7 (5.3)	9.152 (0.256)	6.213 (0.050)	3.259 (0.146)	253.3 (4.85)	20.5 (0.42)
IT1	61.3	12243.6 (306.2)	124.2 (1.8)	122.9 (1.6)	10.6 (2.1)	2873.0 (61.0)	29.2 (0.539)	216.5 (6.4)	9.254 (0.278)	6.081 (0.048)	4.534 (0.155)	283.8 (4.65)	21.5 (0.46)
IT2	43.8	12225.5 (373.9)	123.2 (2.1)	123.0 (2.1)	10.6 (1.9)	2856.5 (83.1)	28.3 (0.655)	227.3 (21.1)	8.722 (0.285)	6.144 (0.073)	4.526 (0.229)	278.5 (6.68)	21.4 (0.50)
NE1	30.2	11506.7 (518.4)	123.7 (2.4)	116.3 (3.0)	24.5 (5.4)	2805.3 (79.5)	26.3 (0.759)	189.8 (6.6)	9.246 (0.415)	6.331 (0.077)	3.655 (0.226)	255.4 (7.02)	19.6 (0.61)
NE2	6.7	9886.3 (313.8)	116.1 (2.8)	107.5 (1.3)	18.3 (7.8)	2622.3 (100.1)	27.1 (0.552)	185.7 (7.2)	8.309 (0.740)	6.088 (0.229)	3.834 (0.409)	270.7 (14.15)	22.1 (1.30)
NE3	11.9	10505.7 (591.7)	118.3 (2.0)	113.3 (4.0)	23.5 (10.9)	2631.6 (129.0)	26.8 (0.652)	193.8 (7.6)	9.540 (0.635)	5.990 (0.080)	3.886 (0.380)	262.6 (4.20)	20.2 (0.73)
SP1	48.7	3212.7 (311.1)	63.5 (2.4)	64.5 (2.4)	35.6 (8.4)	689.3 (51.0)	25.6 (0.701)	183.5 (7.6)	8.834 (0.396)	6.388 (0.070)	1.045 (0.133)	182.7 (6.11)	10.4 (0.45)
SP2	55.4	2643.9 (163.7)	58.8 (1.7)	57.9 (1.7)	27.0 (5.3)	630.4 (45.2)	24.4 (0.675)	174.1 (5.9)	9.141 (0.296)	6.380 (0.061)	0.889 (0.066)	163.4 (5.34)	9.6 (0.39)

N = mean number of samples per trait
LA = leaf area (square mm)
LL = leaf length (mm)
LW = leaf width (mm)
LL_LW = ratio of leaf length to width (x10^4)
SLA = specific leaf area (mg/sq. mm)
Stomata = number of stomata observed on abaxial leaf surface
SD = abaxial stomatal density (number per sq. mm)
SI = abaxial stomatal index (# stomata / total # epidermal cells)
CA = average area of 10 pavement cells (x10^4, sq. mm)
CNPL = calculated number of cells per leaf (x10^-7)
Height = Height of ramet, cm
Circum = circumference of ramet (mm)

Pairwise correlations among 12 morphological traits assessed in a common garden study of *P. nigra*. Significance determined using one-tailed tests.

	Leaf length	Leaf width	LL:LW	Specific leaf area	Cell area	Cell number	Stomatal number	Stomatal density	Stomatal index	Height	Circ.
Leaf area	0.962***	0.964***	-0.236***	0.913***	-0.195***	0.894***	0.363***	0.321***	0.123**	0.736***	0.793***
Leaf length	---	0.938***	-0.201***	0.893***	-0.200***	0.872***	0.351***	0.316***	0.121**	0.733***	0.790***
Leaf width		---	-0.280***	0.899***	-0.209***	0.859***	0.339***	0.277***	0.085*	0.744***	0.815***
Length:width			---	-0.235***	-0.206***	-0.170***	-0.075	-0.159**	-0.161**	-0.312***	-0.234***
Specific leaf area				---	-0.197***	0.836***	0.363***	0.315***	0.144**	0.769***	0.839***
Cell area					---	-0.404***	-0.455***	-0.297***	-0.090*	-0.089*	-0.192***
Cell number						---	0.483***	0.449***	0.192***	0.686***	0.732***
Stomatal number							---	0.724***	0.402***	0.282***	0.320***
Stomatal density								---	0.579***	0.300***	0.279***
Stomatal index									---	0.124**	0.110*
Height										---	0.880***
Circ.											---

*** P<0.001
** P<0.01
* P<0.05

Table 5. Correlations between genetic (Rousset 1997), morphological (from DFA), and log geographic (km) distances among 13 populations of *P. nigra* tested with simple and partial Mantel tests.

**Comparison**	***r***	**P-value**
Simple Mantel tests		
Genetic, Geographic	0.577	<0.001
Morphological, Genetic	0.476	<0.001
Partial Mantel tests		
Genetic, Geographic| Morphological (IBD)	0.440	<0.001
Morphological, Genetic|Geographic (IBA)	0.257	0.012
